# Haemostatic chitosan coated gauze: in vitro interaction with human blood and in-vivo effectiveness

**DOI:** 10.1186/s40824-015-0044-0

**Published:** 2015-11-02

**Authors:** M. Pogorielov, O. Kalinkevich, V. Deineka, V. Garbuzova, A. Solodovnik, A. Kalinkevich, T. Kalinichenko, A. Gapchenko, A. Sklyar, S. Danilchenko

**Affiliations:** Sumy State University, 2, R-Korsakova Street, Sumy, 40018, Ukraine Ukraine; Institute of Applied Physics, 58, Petropavlovskaya Street, 40000 Sumy, Ukraine; Sumy State Pedagogical University, 87, Romenska Street, 40000 Sumy, Ukraine

**Keywords:** Chitosan, Haemostatic dressing, Erythrocytes, Platelets

## Abstract

**Background:**

Chitosan and its derivates are widely used for biomedical application due to antioxidative, anti-inflammatory, antimicrobial and tissue repair induced properties. Chitosan-based materials also used as a haemostatic agent but influence of different molecular weight and concentration of chitosan on biological response of blood cells is still not clear.

The aim of this research was to evaluate interaction between human blood cells and various forms of chitosan-based materials with different molecular weight and chitosan concentration and prove their effectiveness on in-vivo model.

**Methods:**

We used chitosan with molecular weight 200, 500 and 700 kDa and deacetylation rate 80-82 %. For chitosan impregnation of gauze chitosan solutions in 1 % acetic acid with different concentrations (1, 2, 3, 5 %) were used. We used scanning electron microscopy to obtain information about chitosan distribution on cotton surface; Erythrocyte agglutination test and Complete blood count test – for evaluation of interaction between blood cells and chitosan-based materials with different compound. In-vivo studies was performed in 20 Wistar rats to evaluate effectiveness of new dressing.

**Results:**

Our data shown that chitosan can bind erythrocytes in concentration-depend manner that does not depend on its molecular weight. In addition, chitosan-based materials affect selectively human blood cells. Composition of chitosan with cotton materials does not change erythrocyte shape and does not cause agglutination.

**Conclusions:**

Сotton-chitosan materials have higher adhesive properties to platelets that depend on molecular weight and concentration of chitosan. These materials also change platelets’ shape that probable is one of the most important mechanisms of haemostatic effect. In-vivo studies have shown high effectiveness of 2 % 200 kDa chitosan for stop bleeding from arteries of large diameter.

## Background

Adequate haemostasis after trauma and during surgical operation is a big challenge in modern medicine. About 40 % traumatic and more than 90 % of combat deaths took place in pre-hospital settings. And about 50 % from these deaths have been reported due to massive blood loss [1]. Sauaia A. reported 80 % of civilian trauma fatalities within the United States caused by uncontrollable haemorrhage [27]. Also, haemorrhage in trauma patients is the leading cause of reoperation [8].

Topical haemostatic treatment was applied since ancient time. They used herbs, mixture of wax, grease and barley and also animal hides mixed with hot sand to stop bleeding [7]. Advances in biotechnology have resulted in explosive growth of topical haemostatic agents in the last two decades [18, 21, 22]. At present, the scientific community is actively searching for materials which effectively stop the bleeding, but without side effects. Particular attention is paid to the materials based on natural polymers (collagen, chitosan and others) [14, 23].

**Fig. 1:**
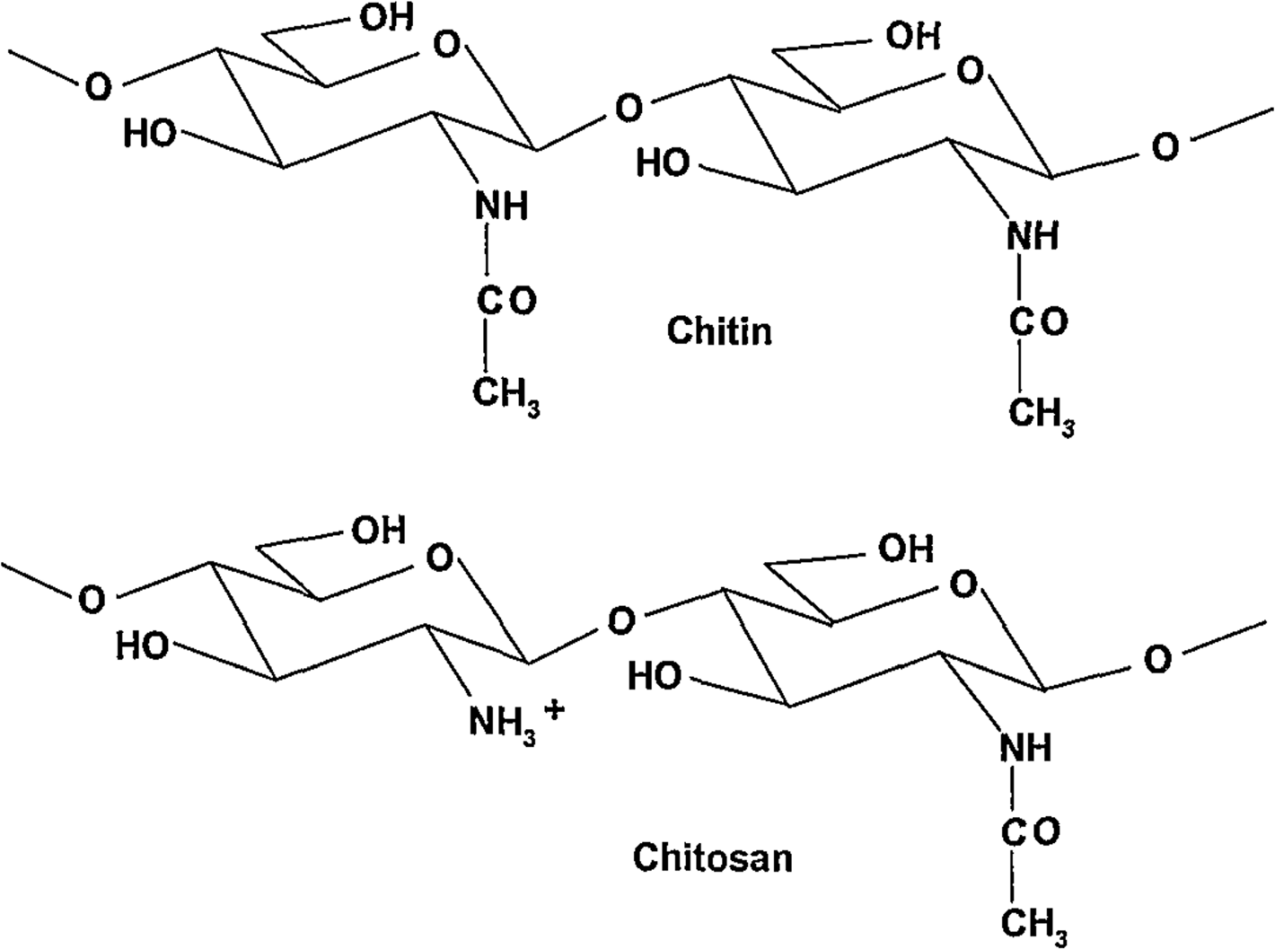
Chitin and chitosan

Chitin and chitosan haemostatic dressing are most promising due to effective blood stop and possible additional properties like antibacterial and stimulatory to regeneration. Chitosan is a linear, semi-crystalline polysaccharide composed of (1–4)-2-acetamido-2-deoxy-b-D-glucan (N-acetyl D-glucosamine) and (1–4)-2-amino-2-deoxyb-D-glucan (D-glucosamine) units [26]. It is not extensively present in the environment – however, it can be easily derived by the partial deacetylation of a natural polymer chitin (Fig. 1). To be named “chitosan”, the deacetylated chitin should contain at least 60 % of D-glucosamine residues [4]. Molecular weight of chitosan typically ranges from 300 to 1000 kDA, depending on the source and preparation [15].

Chitin and chitosan are biocompatible polymers but there are some evidences that chitosan is more cytocompatible in vitro than chitin [25]. While the number of positive charges increases, the interaction between cells and chitosan increases as well, which tends to improve biocompatibility [5]. Materials, based on chitosan have no allergic effect in living body and not toxic [20].

The most studied chitosan-based hemostatic dressings for today are Celox and HemCon. Both dressing were evaluated experimentally and show high effectiveness [3, 3, 13, 16].

Various forms of chitosan-based haemostatic materials (foams, sponges, films, etc.) are developed, the production of which is expensive. One of the easiest ways to get haemostatic materials is steeping of an existing cotton (or other) gauze or bandage in chitosan to improve its haemostatic properties.

Both clinical and experimental evaluations of chitosan-based hemostatic dressing suggest their high effectiveness and safety in civil and battlefield application [10, 15, 23]. But still there is no understanding how does molecular weight of chitosan influence haemostatic activity of chitosan-based materials. Also chitosan may be present in different concentration that can change effectiveness and time that need to stop bleeding.

The chitosan could have different molecular weight, deacetylation rate, and could be chemically modified. Also it is possible to prepare different physical forms: gels, foams, films, sponges, etc., that increase the number of potential chitosan-based materials with different physical, chemical and biological properties [9]. Chitosan and its derivates are widely used for biomedical application due to antioxidative, anti-inflammatory, antimicrobial and tissue repair induced properties [29]. Chitosan derivates are used as haemostatic agents [10, 24]. A lot of commercial materials are used with this purpose, such as HemCon, Chitoseal, Celox, Tegasorb, and QuikClot. They proved high effectiveness in experimental model as well as in civil and combat trauma. But the influence of different molecular weight and concentration of chitosan on biological response of blood cells is still not clear [2, 19].

The aim of this research was to evaluate interaction between human blood cells and various forms of chitosan-based materials and prove their effectiveness on in-vivo model

## Methods

### Preparation of haemostatic dressings with chitosan

We used chitosan with molecular weight (MW) 200, 500 and 700 kDa and deacetylation rate 80-82 % (Bioprogress, Moscow) in our research For impregnation of gauze chitosan solutions in 1 % acetic acid with different concentrations (1, 2, 3, 5 % w/w) were used. Using these solutions we made following materials with potentials haemostatic activities:

#### Gauze-chitosan dressing *(G-Ch)*

Ten layers of gauze were soaked by chitosan solution in a ratio 1 to 5. Gauze was dried in thermostat during 24 h at temperature 36 °C.

Finally we obtained 3 different groups of samples:

- G-Ch dressing with chitosan MW 200 kDa and chitosan concentration 2 %, 3 % and 5 %;
- G-Ch dressing with chitosan MW 500 kDa and chitosan concentration 2 %, 3 % and 5 %;
- G-Ch dressing with chitosan MW700 kDa and chitosan concentration 1 % and 2 %. (1 % solutions of chitosans with MW 200 and 500 kDa are not enough viscous to made dressings, while 3 % and 5 % solutions of 700 kDa chitosan are too viscous.)

#### Standard gauze dressing (SGD)

Ten layers of standard cotton gauze were used as a control.

All samples were cut to make thin strip with average weight 100 mg. Before experiment all materials were stored in fridge with temperature 4 °C.

### Scanning electron microscope

Scanning electron microscopy was performed using the electron microscope REMMA102 (SELMI, Ukraine) to obtain information about chitosan distribution on cotton surface. To avoid surface charge accumulation in the electron-probe experiment standard cotton dressing and gauze-chitosan dressing were covered with the thin (30–50 nm) layer of silver in the vacuum set-up VUP-5 M (SELMI, Ukraine).

To obtain information about the interaction between blood cells and materials that we used in experiment, after the haemostasis occure we put SCG and G-Ch to 5 % formaldehyde for 1 h, dehydrated samples in ethanol and dried.

### Blood

Three human subjects volunteered to have 80 mL of blood drawn by a registered nurse at the Medical Institute of Sumy State University. The study had been previously approved by the Ethic Committee on Medical Research of Medical Institute of Sumy State University. An appropriate informed consent was obtained from all patients. Subjects were healthy adults in age 20, 22, and 24. 2.5 ml of blood was immediately placed to Becton Dickinson Vacutainers® with 3.6 mg EDTA for complete blood count (CBC) test and with 0.109 М sodium citrate – for coagulogram test. 20 ml of blood was centrifuged to made erythrocyte mass for agglutination test.

### Erythrocyte agglutination test

5 ml of erythrocytes were diluted in 95 ml of saline solution. 0.5 ml of chitosan solution was transferred to “U” bottom 96-well microplate and 0.1 ml of 5 % erythrocyte mass was added. The incubation was made in thermostat at 37 °C during 40 min. Hemagglutination was described using a scale purposed by Stavitsky:

- ++++ compact granular agglutinate;
- +++ smooth mat on bottom of well with folded edges;
- ++ smooth mat on bottom of well. Edges somewhat ragged;
- + narrow ring of red around edge of smooth mat;

- discrete red button in centre of bottom well.

Other presentations were interpreted as undetermined events.

### Blood interaction test

The strips of chitosan-based materials and standard cotton gauze with weight 100 mg was placed to Becton Dickinson Vacutainers® and incubated in thermostat in temperature 36 °C during 10 min. All samples were removed and blood transported to the hematology tests. Untreated blood was used as a control.

#### Sorption of blood

All samples removed from Becton Dickinson Vacutainers® with human blood were weighed to evaluate sorption of blood. It was determined using following formula (1):

1 $$ \mathrm{S} = {\mathrm{W}}_1\hbox{-} {\mathrm{W}}_2 $$

where S – sorption of blood (mg); W_1_ – sample weight after experiment (mg); W_2_ – sample weight before experiment (mg).

#### Complete blood count test

CBC-test and coagulogram test was performed in Medical Centre “Floris”. CBC was carried out on the haematology analyzer CELL-DYN 3700 (ABBOTT, USA) using reagents DIAGON (Hungary). We determined haemoglobin level (HGB, g/L), Red Blood Cells (RBC, T/L), Mean Corpuscular Volume (MCV, fL), RBC Distribution Width (RDW, %), Platelets (PTL, G/L), Mean Platelet Volume (MPV, fL), and Platelet Distribution Width (PDW, %).

Study of blood coagulation was performed on an automated analyzer system ACL 7000 (Instrumentation Laboratory, USA) using reagents RecombiPlasTin 2G and SynthASil (HemosIL, USA). We determined prothrombin time (PT, sec), prothrombin index (PI, %), international normalized ratio (INR, EU), fibrinogen (Fg, gram perliter), and activated partial thromboplastin time (APTT, sec).

### Animals and experiment design

Twenty white laboratory rats (Wistar line) weighing 180–200 g, obtained from Vivarium of Medical Institute of Sumy State University, were used in the study. Experiment design was approved by Ethical Committee of Sumy State University.

The rats were divided into two groups of 10 animals in each to evaluate haemostatic effect of chitosan dressing.

All animals were anesthetized with ketamine (10 mg per 1 kg weigh). Medial surface of the tight were shaved. The access sites in each animal were the superficial femoral artery (SFA). We cut the medial surface of SFA of half from its diameter using the surgical scissors. In first group of animals we apply SGD with standard manual compression (SMC) for control bleeding. Second groups received treatment with G-Ch dressing applied with SMC.

In both groups SMC was provided for 60 s, if the bleeding continued, SMC was applied for an additional 60 s. If haemostasis was not achieved after 10 min of treatment uncontrolled haemorrhage was concluded. After the haemostasis occurred we weighed the SGD and G-Ch to obtain information about the amount of blood loss that was determined using following formula (2):

2 $$ \mathrm{S} = {\mathrm{W}}_1\hbox{-} {\mathrm{W}}_2 $$

where S – blood loss (mg); W_1_ – sample weight after experiment (mg); W_2_ – sample weight before experiment (mg).

### Statistic methods

Data were expressed as means ± standard deviation. Student’s *t*-test on unpaired data was used to assess the statistical significance of the difference between the results obtained from the tested specimens. Statistical significance was assumed at a confidence level of 95 % (*p* < 0.05).

## Results

### Scanning electron microscopy

**Fig. 2:**
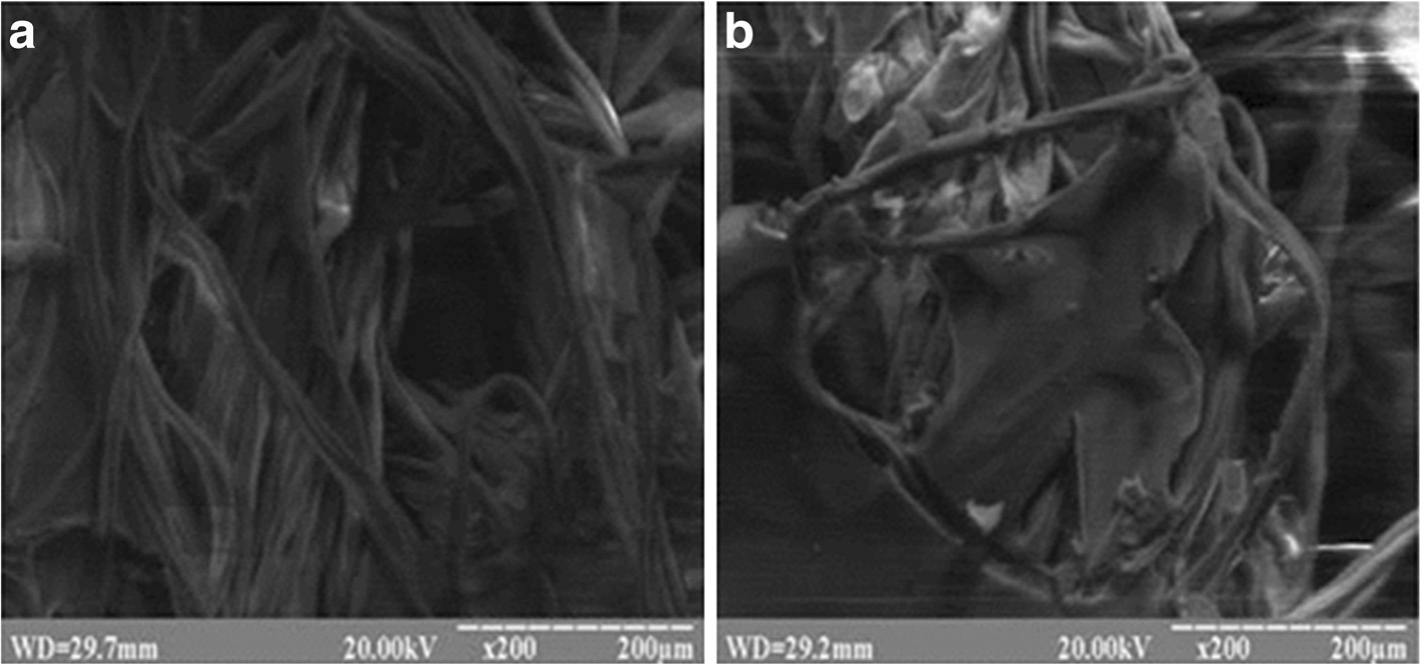
Microstructure of standard cotton dressing (**a**) and Gauze-chitosan dressing (**b**)

Ten layers of SGD made dense network of fibres with pores from 0.1 to 1.0 mm that allow to absorbed blood plasma as well as cells. After soaking of cotton with chitosan solution and drying we can see that cotton fibers are covered with thin layer of chitosan and pores did not close (Fig. 2). It allows blood to contact with chitosan and to be absorbed inside the material.

### Erythrocyte agglutination test

**Fig. 3:**
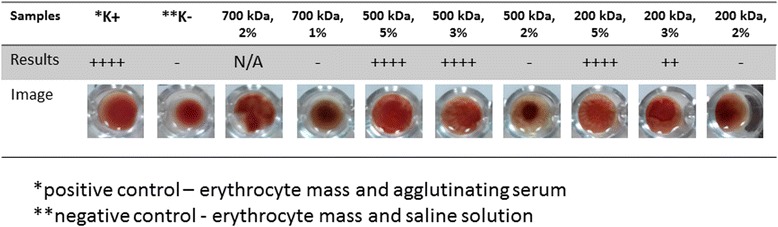
Results of hemagglutination of human erythrocyte in chitosan solutions

Complete hemagglutination index (++++) was present in solution of 3 % and 5 % 500 kDa and 5 % 200 kDa chitosan (Fig. 3). Weak hemagglutination index (++) is in 3 % 200 kDa chitosan solution. Low percentage chitosan solution (1 % and 2 % of all MW) did not cause agglutination. In 2 % 700 kDa chitosan solution we observed undetermined results due to high solution viscosity.

### Sorption of blood

**Fig. 4:**
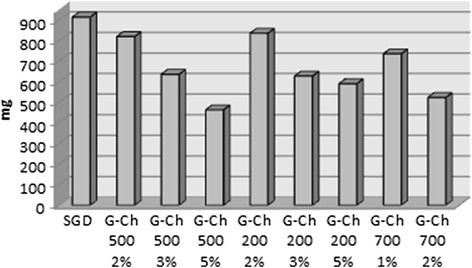
Blood sorption by the SGD and chitosan-based materials in 10 min after blood incubation

SGD, 2 % G-Ch with MW 200 kDa, 500 kDa and 1 % G-Ch with MW 700 kDa absorbed the same amount of blood: from 735 to 913.3 g (Fig. 4). 3 % and 5 % G-Ch with MW500 kDa absorbed significant less blood compared with the SGD and 2 % 500 kDa G-Ch dressing. Also 3 % G-Ch with MW 500 kDa absorbed significant more blood compared to the 5 % ones. Blood sorption by 3 % and 5 % G-Ch with MW 200 kDa is significantly less compared to the SGD and 2 % 200 kDa G-Ch dressing. But there is no significant difference between 3 % and 5 % 200 kDa G-Ch materials. Also we did not see difference between dressing with the same percentage of chitosan but different MW.

### CBC-test

Blood coagulation test did not show any changes of coagulogramm and difference between SGD and chitosan-based materials.

**Fig. 5:**
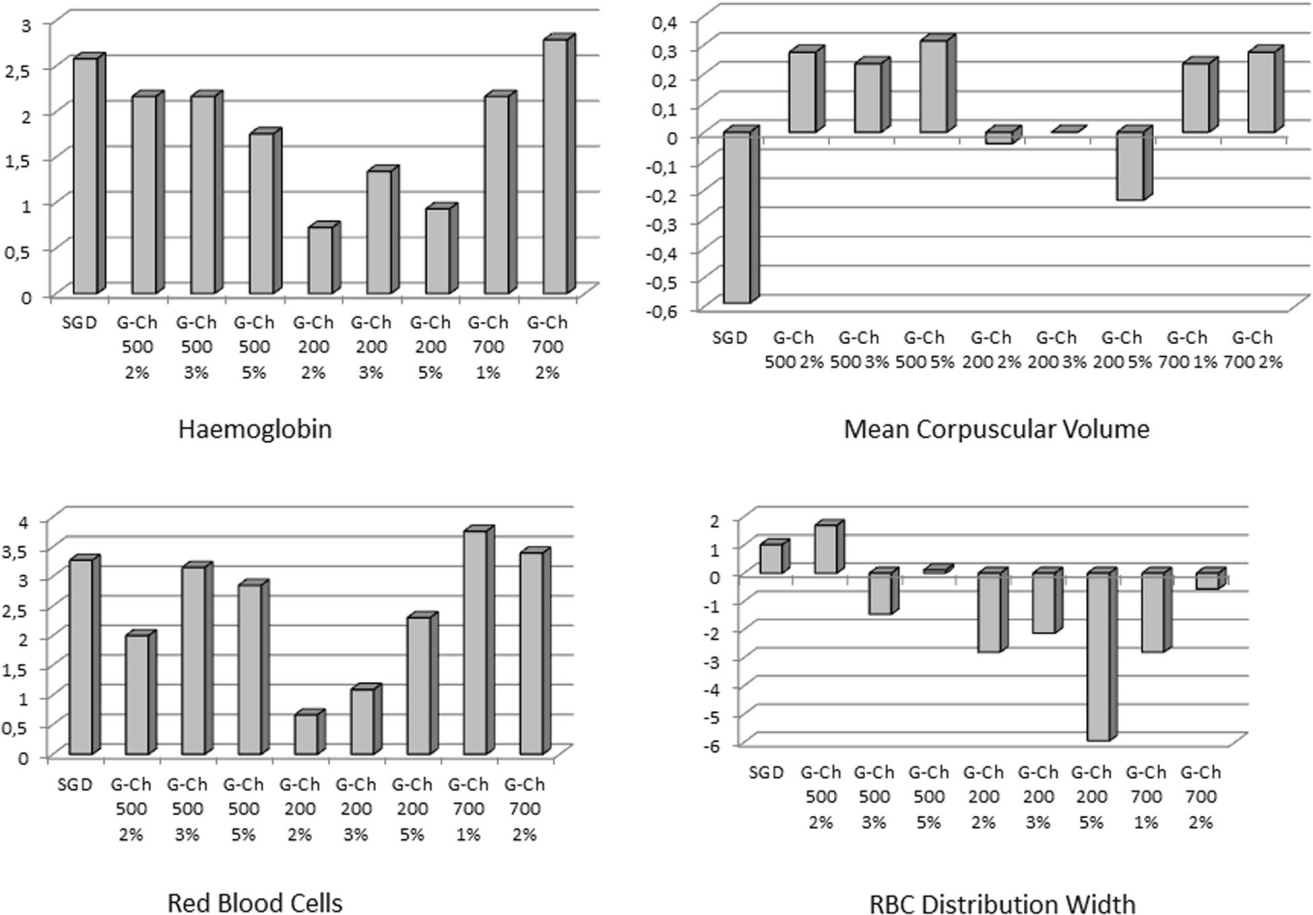
Difference (in %) of HGB, RBC, RDW, and MCP between the control untreated blood (zero line) and blood that interacted with chitosan-based materials

During the CBC-test we focused on Red Blood Cells and Platelets parameters. Current experiment did not show any significant differences between control (untreated blood) and blood that interacted with chitosan-based materials in RBC concentration, haemoglobin level, RBC Distribution Width, and Mean Corpuscular Volume (Fig. 5).

**Fig. 6:**
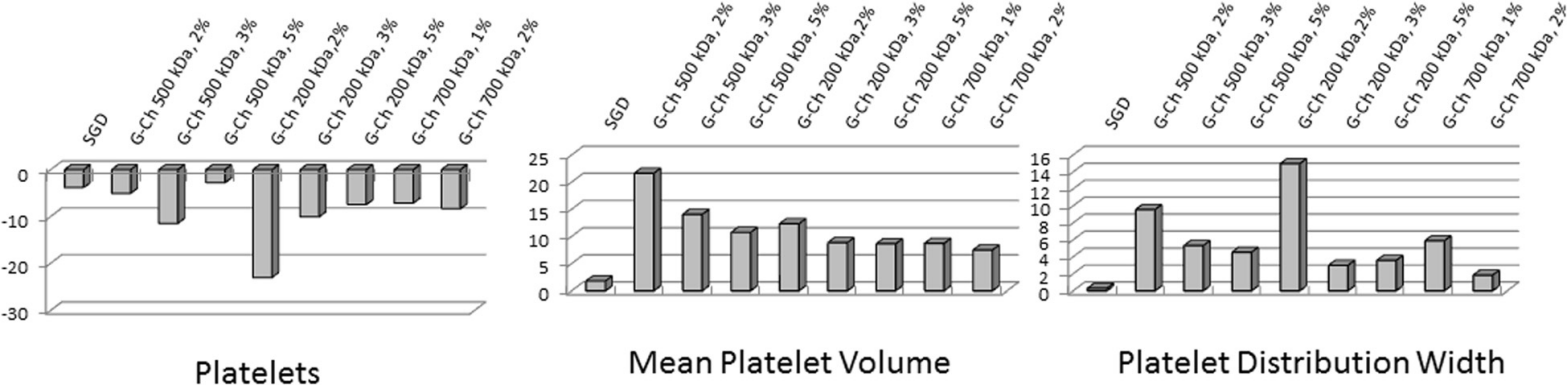
Difference (in %) of platelets, MPV, and PDW between the control untreated blood (zero line) and blood that interacted with chitosan-based materials

Compared with the RBC, amount of platelets and its parameters significantly changed depending on MW, percentage of chitosan in solution and type of material (Fig. 6). All types of G-Ch dressings made from 200 kDa MW chitosan significantly decreased platelets amount in blood in 10 min after incubation. Materials made from 2 % chitosan solution have the strongest effect: platelets level decreased compared with the control group by 23.03 % (*p* ≤ 0.001). Samples of chitosan MW 700 kDa significantly decreased platelets level, but the difference was not more than 8.33 % (*p* = 0.023). Materials made from 500 kDa chitosan did not change platelets level except the samples of 3 % solution that caused significant depression of cell concentration in blood.

Our data show that all types of G-Ch dressing affected Mean Platelet Volume. Materials made from chitosan with MW 500 kDA caused more significant augmentation of platelets volume compared to 200 and 700 kDa samples. Most prominent difference we can see for materials made from 2 % solution of 500 kDa MW chitosan: it is equal to 21.63 % (*p* ≤ 0.001). Platelet Distribution Width changed only in blood samples that interacted with 2 % chitosan with MW 200 and 700 kDa. All other samples did not affect this parameter.

### In-vivo experiment

For in-vivo experiment we have used G-Ch with MW 200 kDa and concentration of 2 % due to its in-vitro effectiveness. We see the effectiveness of G-Ch dressing in 9 form 10 animals compare the SGD that stop bleeding in 20 % from all cases (Table 1).

**Table 1:** Effectiveness of haemostasis during the in-vivo experiment

Group	N	Complete haemostasis up to 10 min	% of effectiveness
SCG	10	2	20
G-Ch	10	9	90

**Fig. 7:**
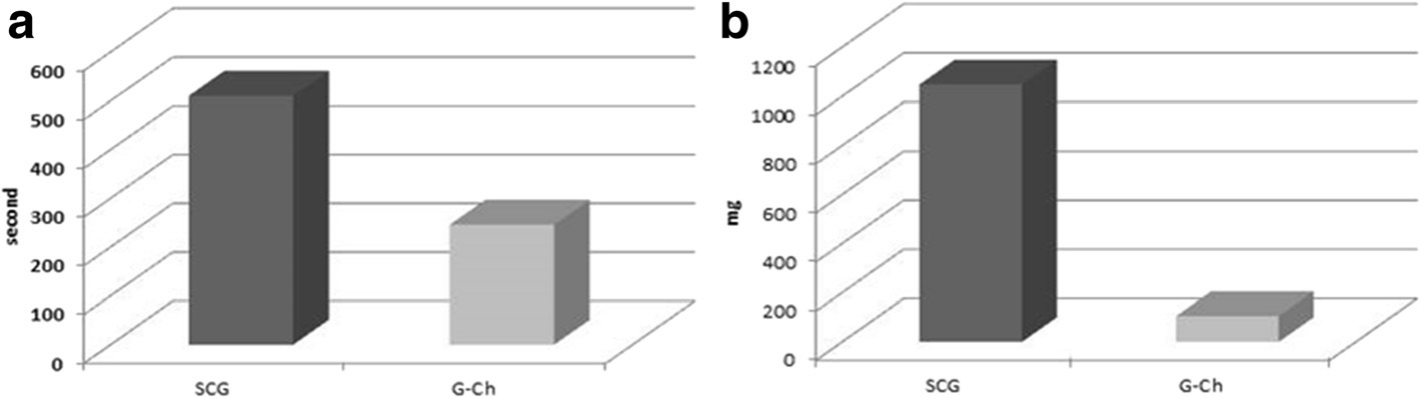
Time of complete haemostasis (**a**) and blood loss (**b**) after application of SGD and G-Ch dressings

Time of haemostasis and amount of blood loss were determined in survived animals only (Fig. 7). Time of haemostatis after application of G-Ch (256 ± 24.71 s) was in two times less compare the SGD (510 ± 11.34) seconds. Animals that were treat using G-Ch loss 105 ± 17.32 mg of blood, but application of SGD lead massive blood loss - 1046 ± 164.43 mg during the haemostatic procedure.

**Fig. 8:**
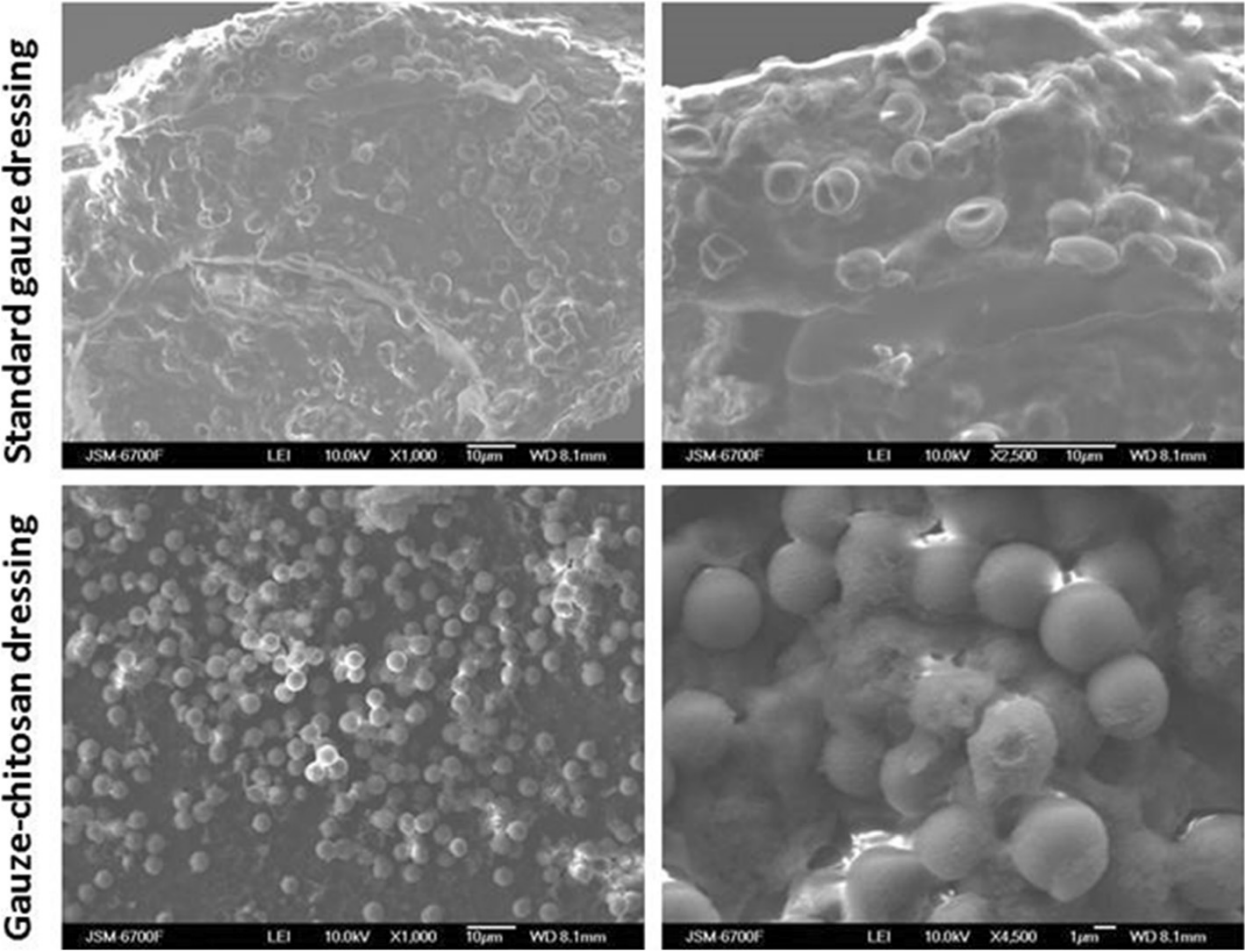
Scanning electron microscopy of SGD and G-Ch after the successful haemostasis

Scanning electron microscopy show that blood cells pure adhere to SGD. We can see (Fig. 8) that erythrocytes slightly spread on dressing surface, have usual shape and do not aggregate.

Compare to this results, G-Ch dressing adhere a lot of red blood cells. Erythrocytes imbed to the loose substance, that is probably chitosan on gauze surface. We can see that almost all cells change their shape and looks like spherocyte.

## Discussion

The chitosan could have different molecular weight, deacetylation rate, and could be chemically modified. It is a cationic polymer and can bind red blood cells due to their anionic nature [17]. Hemagglutination assay in current experiments shows that erythrocyte agglutination strongly depends on chitosan concentration in solution and doesn’t significantly depend on its molecular weight. All samples with chitosan concentration from 3 % to 5 % show agglutination, while 1 % and 2 % do not. Erythrocyte aggregation in chitosan solution as well as films and chitosan nanoparticles is presented in some research but they did not show relations between chitosan concentration and agglutination effect. Moreover, S.B. Rao has shown that 2 % of chitosan nanoparticle-based film causes hemagglutination [24]. In our research we observed weak agglutination only in 2 % solution of 200 kDa chitosan, other molecular weight chitosans with same concentration did not cause erythrocyte aggregation. Probably, high concentration chitosan solutions have more protonated groups and thus more chances to bind with erythrocyte wall. But extremely high concentration increases the viscosity of chitosan solution and decreases agglutination effect. Our experiments show that high molecular weight increases viscosity and it is simpler to made high concentration solution from low molecular weight chitosan with same agglutination effect.

Sorption properties are important for a haemostatic agent due to possibility of fast plasma sorption and increasing concentration of blood cells, especially RBC and platelets, near injured vessel. Our data proved that materials made from cotton and middle and high MW chitosan absorbed the same amount of blood as a standard cotton dressing. Low MW chitosan significantly decrease sorption properties of cotton dressing.

CBC test did not show difference in RBC number as well as their volume and width. Some researchers showed that chitosan-based materials can change erythrocyte shape but they did not prove RBC adhesion on chitosan. Klokkevold et al. revealed that erythrocytes from anesthetized rabbits lost their typical biconcave morphology and appeared to have an unusual affinity towards one another [11]. Kind et al. did not see any RBC change after the contact with chitosan material [10]. Current experiments shown that influence on RBC depends on form of chitosan: solution with high concentration caused erythrocyte coagulation but chitosan-based dressing did not affect normal human RBC.

Platelets are the essential mediators that trigger the mechanical pathway of the coagulation cascade. Previous researches have shown that platelets adhered to chitosan-based materials in time and concentration-dependent manner [6]. Shen et al. discovered that chitosan induced platelet adhesion and aggregation by noting that the level of aggregation was related to the concentration of the platelets in the plasma [28]. Our data shown that level of platelets significantly decrease in blood in 10 min of chitosan interaction that proved their adhesion to chitosan-based material. The adhesion depends on molecular weight and concentration. Low and high MW chitosan-cotton dressing significantly decrease platelets level while middle MW have no same affect. High concentration of chitosan (3 % and 5 %) decrease platelets adhesion. Platelets shape (volume and width) changed in all chitosan-cotton groups but the most significant changes were observed for 500 kDa chitosan samples. Probably, Low and High molecular weight chitosan adsorbed more platelets and possibly changed their shape, and we did not see it in the remnant of blood. Middle molecular weight chitosan did not adsorb a lot of cells but significantly changed their shape. The same morphological parameters of platelets were observed during the clot formation that proved haemostatic potential of chitosan-based materials. The mechanisms that induce platelets adhesion and activation by chitosan are still not understood completely. There are some evidences that chitosan can activate Glycoprotein IIb/IIIa and discharge thromboxane A2/ADP and these signals elevate platelet spreading and strengthen the stability of adhesion [28].

In-vivo results shown 90 % effectiveness of chitosan-based dressing compare the 20 % in control group with SGD. In several studies, chitosan based materials provides up to 100 % effectiveness during different injury models [12, 21]. It is very important the time of haemostasis and amount of blood loss during treatment. Our data shown that application of gauze-chitosan dressing decrease these parameters in 2 and 10 times compare the SGD. Koksal O. and coauthors shown that Celox stop bleeding in rats that used anticoagulants after 90 s compare the standard manual compression that was ineffective [11]. Other chitosan-based dressing – HemCon, stop bleeding after 6.94 ± 3.9 min in SFA trauma in ship model.

To summarize our results and literature data about chitosan action on blood coagulation and haemostasis we propose mechanism of action of our dressing made by the impregantion of cotton gauze with chitosan solution.

**Fig. 9:**
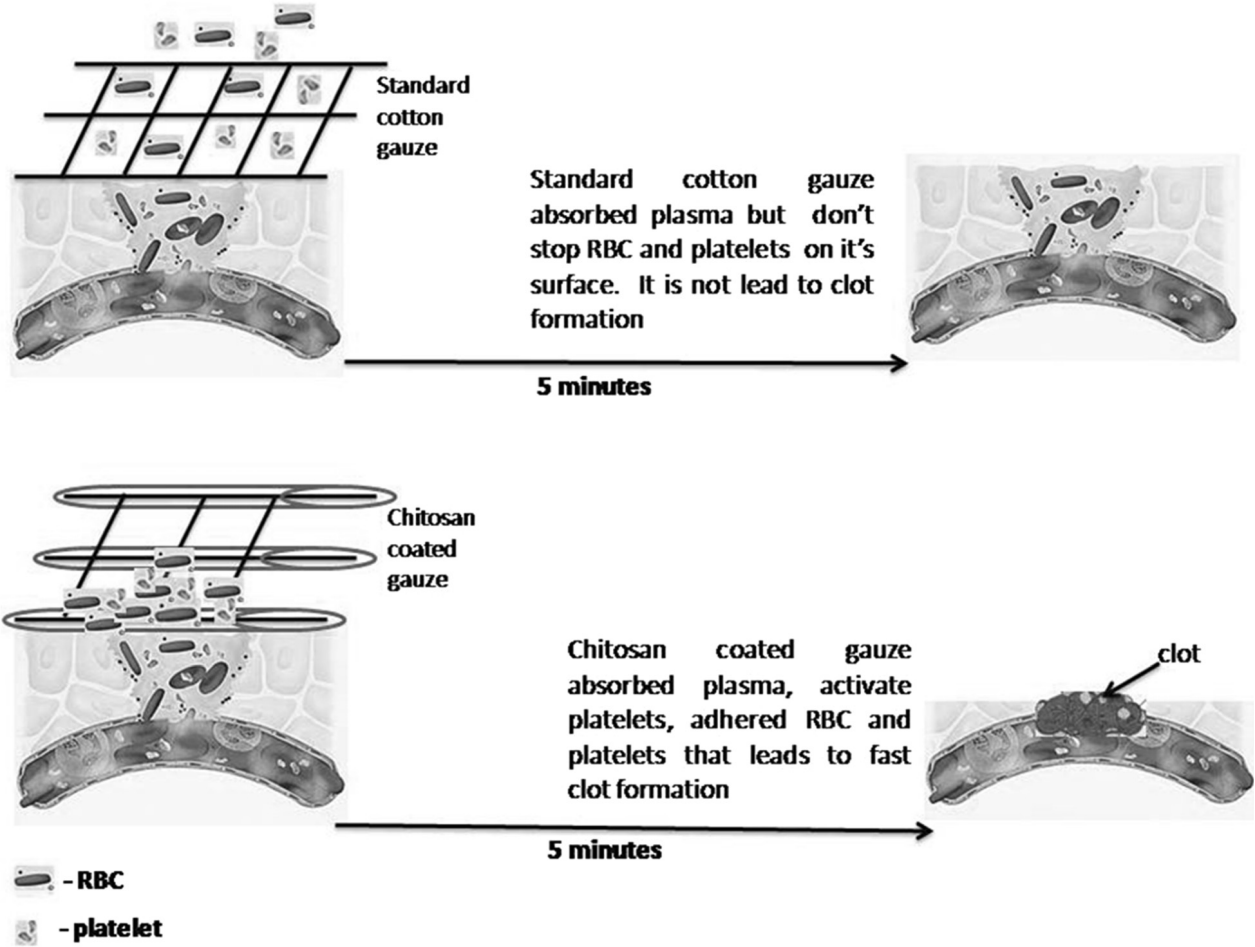
Scheme of clot formation after application of gauze-chitosan dressing

Standard cotton gauze after application sorbed plasma and blood cells but the last one did not adhere to dressing. In this case, gauze act as a conductor for blood and clot did not form on injured artery. Chitosan adhered erythrocytes and platelets on gauze surface that turn to the injury. Also chitosan can absorbed fibrin that form a network above the injured artery. It is facilitate clot formation and prevent further bleeding (Fig. 9).

## Conclusion

Chitosan can bind erythrocytes in concentration-depend manner that does not depend on its molecular weight. In addition, chitosan-based materials affect selectively human blood cells. Composition of chitosan with cotton materials does not change erythrocyte shape and does not cause agglutination. But cotton-chitosan materials have higher adhesive properties to platelets that depend on molecular weight and concentration of chitosan. These materials also change platelets’ shape that probable is one of the most important mechanisms of haemostatic effect. In-vivo studies show high effectiveness of G-Ch dressing during the trauma of large vessel and prove blood cells changes during the chitosan-induced haemostasis.
